# Supplementary material for: Splice-Junction-Based Mapping of Alternative Isoforms in the Human Proteome
Source: Cell Rep. Author manuscript; Available in PMC 2020 Jan 15. (PMC6961840; doi:10.1016/j.celrep.2019.11.026)
Supplement: 2 [file NIHMS1546469-supplement-2.zip › DF1/PXD009737/TestisLysC/TestisLysC_29_SRSF11_IDELRLFPPEK.pdf]

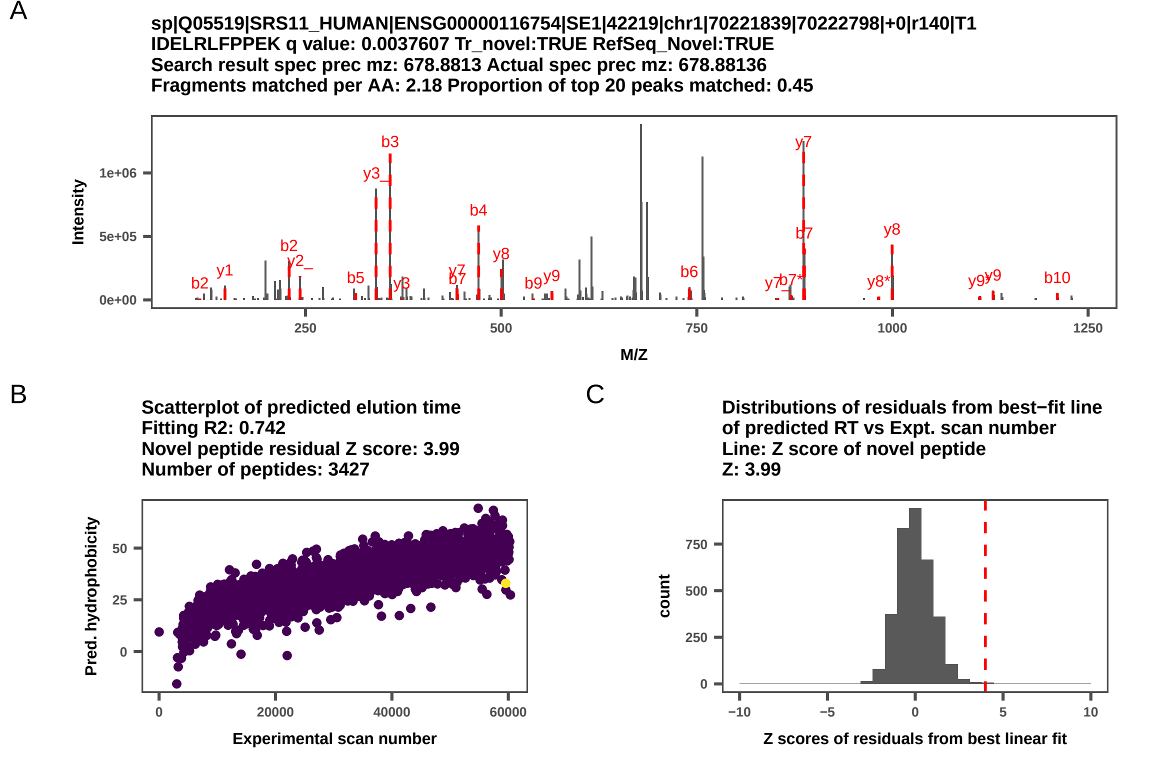
A
sp|Q05519|SRS11_HUMAN|ENSG00000116754|SE1|42219|chr1|70221839|70222798|+0|r140|T1
IDELRLFPPEK q value: 0.0037607 Tr_novel:TRUE RefSeq_Novel:TRUE
Search result spec prec mz: 678.8813 Actual spec prec mz: 678.88136
Fragments matched per AA: 2.18 Proportion of top 20 peaks matched: 0.45
Intensity
1e+06
5e+05
0e+00
b2
y1
b2
y2_
b3
y3_
b5
y3
b4
y7
b7
y8
b9
y9
b6
y7
b7*
y7
b7
y8
y8*
y9*
y9
b10
250
500
750
1000
1250
M/Z
B
Scatterplot of predicted elution time
Fitting R2: 0.742
Novel peptide residual Z score: 3.99
Number of peptides: 3427
Pred. hydrophobicity
50
25
0
0
20000
40000
60000
Experimental scan number
C
Distributions of residuals from best−fit line
of predicted RT vs Expt. scan number
Line: Z score of novel peptide
Z: 3.99
count
750
500
250
0
−10
−5
0
5
10
Z scores of residuals from best linear fit
